# Developing international open science collaborations: Funder reflections on the Open Science Prize

**DOI:** 10.1371/journal.pbio.2002617

**Published:** 2017-08-01

**Authors:** Elizabeth Kittrie, Audie A. Atienza, Robert Kiley, David Carr, Aki MacFarlane, Vinay Pai, Jennifer Couch, Jared Bajkowski, Joseph F. Bonner, Daniel Mietchen, Philip E. Bourne

**Affiliations:** 1 National Institutes of Health, Bethesda, Maryland, United States of America; 2 ICF, Rockville, Maryland, United States of America; 3 Wellcome Trust, London, England, United Kingdom; 4 Howard Hughes Medical Institute, Bethesda, Maryland, United States of America; 5 University of Virginia, Charlottesville, Virginia, United States of America

## Abstract

The Open Science Prize was established with the following objectives: first, to encourage the crowdsourcing of open data to make breakthroughs that are of biomedical significance; second, to illustrate that funders can indeed work together when scientific interests are aligned; and finally, to encourage international collaboration between investigators with the intent of achieving important innovations that would not be possible otherwise. The process for running the competition and the successes and challenges that arose are presented.

## Introduction

Open data and open science can confer benefits to researchers, including increases in citation, media attention, and potential collaborations [1]. While open access to scientific publications has increasingly received attention in the research literature [2], information on open data, especially in terms of encouraging international collaborations that leverage this content to address key biomedical and public health issues, is not well addressed. In most biomedical fields, data sharing does not normally happen before the research is concluded, while open science refers to sharing the research process and its outcomes on an ongoing basis [3]. There are some excellent examples of open data and open science projects that encourage crowdsourcing of solutions to scientific problems (e.g., in malaria research [4]), but overall adoption of open research practices has been limited, particularly in biomedical research. We argue that open science and international collaboration are needed to address global health issues, and we describe a funding mechanism that was successful in encouraging that.

### Context for setting up the Open Science Prize

To encourage international partnerships in leveraging open research data, the National Institutes of Health (NIH), Wellcome Trust (WT), and Howard Hughes Medical Institute (HHMI) partnered on funding the Open Science Prize (OSP). The importance of open data and open science for the NIH is reflected in its strategic plan [5], which states, “NIH will serve as a focal point for catalyzing this historic research opportunity, continuing to leverage its roles as an influential convener and major funding agency to encourage rapid, open sharing of data and greater harmonization of scientific efforts.” WT has long championed open scientific research [6], including open access to publications and, more recently, the sharing of research data sets and computer code. Similarly, HHMI has a long-standing policy that strongly encourages their investigators to make publications publicly available [7] and make data and other research materials available to other scientists [8]. To our knowledge, the OSP is the first multifunder initiative focusing on international collaborations to develop open science technologies that enable open digital content to be discovered, accessed, and reused in ways that will advance research, spark innovation, and generate new societal benefits.

The OSP utilized a prize competition as a funding mechanism (see announcement: https://www.federalregister.gov/documents/2015/10/20/2015-26392/announcement-of-requirements-and-registration-for-the-open-science-prize). The prize was open to any solution that exhibited the benefits of making research outputs open and usable and demonstrated new and innovative ways of enabling this. For NIH, the America Creating Opportunities to Meaningfully Promote Excellence in Technology, Education, and Science Reauthorization Act of 2010 (also known as America Competes Act) provided United States funding agencies the authority to provide prizes and awards [9]. The use of prize competitions as a funding mechanism has several advantages over traditional funding mechanisms (e.g., grants, contracts). Awards are only given to successful solutions, solvers can include anyone with the skills and knowledge to address the specified solution, and the time and expense to run a prize competition is typically much less than a traditional funding mechanism. For example, traditional NIH grants such as the Small Business Innovation Research and Small Business Technology Transfer or Research Project Grant (RO1) funding mechanisms tend to favor academic researchers (investigator background and research environment are 2 criteria in the peer-review process) and typically take a significant amount of time to produce results. The OSP was open to all interested solvers, and the entire process was completed in 2 years from conceptualization to awarding of the grand prize. Finally, co-funding collaborative projects with international funders using traditional government funding mechanisms can be complicated compared with prize competitions. The OSP provided an administrative mechanism through which 3 agencies could share funding responsibilities and in-kind resources. However, competitors and winners were predominantly based in academia. This can perhaps be attributed to the fact that the 3 funding agencies mostly fund academics. Future competitions would need extensive public relations to broaden the appeal.

There are several aspects of the OSP that were notable. First, international collaborations among solvers were required, with at least 1 team member based in the US and 1 team member based outside of the US. Second, the prize competition was structured in 2 phases: Phase I sought the most innovative and promising ideas and proposed open science tools (6 awards; US$80,000 each) and Phase II provided a grand prize award (US$230,000) for the best prototype of the Phase I finalists. Thus, not only could a diverse set of ideas come forth, but initial feedback could be given to the most promising ideas in the prototype phase. Third, an innovative funding model was incorporated for the OSP. NIH provided funds to the US member(s) of the teams selected for the awards per requirements in the America Competes Act, and WT and HHMI provided funds to the non-US member(s). Fourth, in an effort to invite broad input on the innovations, a group of advisors (see acknowledgments) with expertise in open science, health information technology, and/or biomedical research methods were selected to provide comments on the submitted applications and prototypes. In addition, the public was invited during a 5-week period to vote and comment on their favorite prototypes of the 6 finalists during Phase II of the competition. Public comments are posted on the OSP website. Fifth, to the extent practical with the resources available, all aspects of the competition were made open. For example, the executive summaries of all submissions were made public on the OSP website, as were the Phase II applications of the 6 winning teams. Some of the teams had also drafted or posted their Phase I submissions in the open, even though this was not required (see GitHub site).

### OSP results

**Fig 1:**
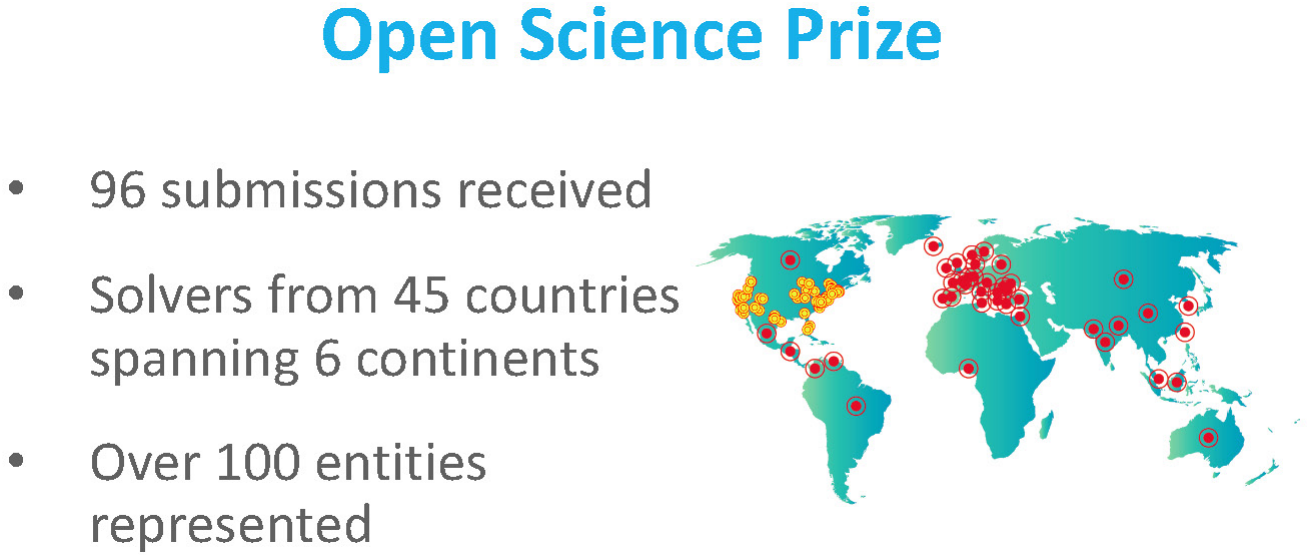
The 6 Phase I finalists were: (a) “OpenAQ: A Global Community Building the First Open, Real-Time Air Quality Data Hub for the World,” providing real-time information on poor air quality by combining data from across the globe, (b) “OpenTrialsFDA: Making Unbiased Clinical Trial Data Accessible,” enabling better access to drug-approval packages submitted to and made available by the Food and Drug Administration, (c) “Open Neuroimaging Laboratory,” advancing brain research by enabling collaborative annotation, discovery, and analysis of brain-imaging data, d) “Fruit Fly Brain Observatory,” allowing researchers to better conduct modeling of mental and neurological diseases by connecting data related to the fly brain, (e) “MyGene2: Accelerating Gene Discovery via Radically Open Data Sharing,” facilitating the public sharing of health and genetic data through integration with publicly available information, and (f) “Real-Time Evolutionary Tracking for Pathogen Surveillance and Epidemiological Investigation,” permitting real-time analysis of emerging epidemics, such as Ebola, Middle East Respiratory Syndrome Conavirus, and Zika.

The OSP received 96 submissions for Phase I of the competition, with entries coming from 45 countries spanning 6 continents (Fig 1). Phase I applications were judged on the following criteria: advancement of open science, impact, innovation, originality, technology viability, and resource feasibility. Based on these criteria, 6 finalists were selected to progress to Phase II of the competition, in which they were provided funds to develop prototypes. The teams were invited to provide live demonstrations of their prototypes at a public Open Data Science Symposium organized by NIH. The teams were asked to develop written narratives summarizing progress to date and addressing key factors such as innovation, utility, feasibility and technical merit, and development and sustainability plan for their prototypes. These summaries, along with videos that many of the teams created, were publicly displayed on the OSP website, and the public was invited to cast their votes and provide input. During Phase II, 3,730 votes were received from voters representing 76 countries. The judging criteria for Phase II was similar to the criteria utilized for Phase I, but in Phase II, the emphasis was on the prototype developed as opposed to an anticipatory idea.

Based on the review of all information provided, the funders selected “Real-Time Evolutionary Tracking for Pathogen Surveillance and Epidemiological Investigation” as the grand prize winner of the OSP. “Real-Time Evolutionary Tracking for Pathogen Surveillance and Epidemiological Investigation” was developed by a team from the Fred Hutchinson Cancer Research Center in Washington and the University of Basel, Switzerland. The project uses an online visualization platform (http://www.nextstrain.org) in which the outputs of statistical analyses of viral genome sequences can be used by public health officials for epidemiological insights within days of genome sequences having been obtained from viral pathogen samples taken from patients. Nextstrain.org expanded upon the submitters’ prior work on influenza tracking (nextflu.org) to include many other viral pathogens. Using OSP funds, the team developed detailed phylogeographic analyses of viral movement, created a platform for rapid (real-time) data sharing of pathogen genome data with open source code, and constructed a database and associated parsing scripts to quickly consolidate emerging data from multiple sources.

### Benefits and challenges to public–private partnerships for international health

The OSP demonstrated the benefit of international funding partnerships. On a very practical level, by combining resources, the 3 funding agencies were able to increase the size of the purse prizes. They were also able to obtain administrative efficiencies by sharing in-kind resources such as website design expertise and video captioning services. The funders were able to widen the reach of this initiative because they were able to leverage the outreach capacities and communications outlets of the 3 funding organizations to tap into broader networks of potential solvers. At times of declining budgets, leveraging resources through partnerships can be a key strategy for promoting innovation. The OSP submissions, which involved teams of international solvers, highlighted how global collaboration can be powerful and effective. An example of this is OpenAQ, a platform for aggregating and sharing air-quality data from around the globe, which through the OSP funding was able to coalesce air-quality measurements from 33 countries. According to the team’s Phase II submission, as a result of OSP funding, its data set has grown by a factor of 7 and currently contains 28 million data points, adding roughly 130,000 measurements per day.

The development of new funding models, like the OSP, is important for several reasons. First, there is a need to further incentivize biomedical discovery with open digital content. One prior example of the power of open data—albeit on a massively different scale—is the Human Genome Project, which made data openly available and has generated a 140-fold return on investment [10]. Initiatives like the OSP offer other funding mechanisms besides traditional grants and contracts to encourage collaborations among researchers and nontraditional scientists (e.g., students, programmers, citizen scientists, and anyone with innovative ideas), encouraging them to engage in scientific discovery by leveraging open biomedical data. Second, many health and disease issues (e.g., Zika, H1N1, HIV/AIDS, environmental toxins) pay little attention to national borders and are optimally addressed with collaborative teams. Collecting and disseminating timely data on these global health threats may expedite the saving of lives and curb the spread of diseases. As evidenced with the OSP, having international funding agencies partner on innovative initiatives can facilitate the development of agile tools/technology and cross-national collaborations to address critical biomedical problems. Third, democratizing science can create novel solutions to complex and/or challenging health problems [11]. Some have further suggested that in order to address intractable global problems, global public goods with open data resources should be established [12]. Yet, the long-term financing of these global public goods remains poorly defined. Moreover, whether academic and/or technology organizations consider the development of such open data resources for biomedical issues to be an important criterion for rewarding staff needs further investigation, as it may determine who is willing participate in initiatives like the OSP.

This public–private funding collaboration faced several challenges when developing this joint initiative. Challenges included crafting an initiative that adhered to the policies and regulations of the different funding organizations, aligning the scope of the initiative to address common interests, and the lack of a precedent for this type of initiative encouraging international collaborations to create digital tools and platforms with open biomedical data. Furthermore, each funding agency has its own approval processes, which added additional time to the development of shared documents such as funding announcements or press releases. More importantly, organizational policy differences had to be considered in the creation of the initiative. For example, NIH’s funding authority came from the America Competes Act, which limited the awarding of funds to US citizens or organizations incorporated in the US; WT and HHMI did not have this restriction. Another policy difference is that HHMI extramural-funded investigators have dual roles of academic faculty and HHMI employees, whereas extramural investigators funded by NIH and WT are not employees of the respective funding organizations. All of these issues needed to be considered in the development of this partnership.

While the development of a prototype is an important first step toward deriving value from open data, we found that the US$80,000 Phase I awards we provided to establish proof-of-concept prototypes did not necessarily ensure that the tools could confirm longer-term financial sustainability plans within the time frame of the contest. Some teams were able to leverage the Phase I funding to acquire additional funds. For example, the lead investigator participating in “Real-Time Evolutionary Tracking for Pathogen Surveillance and Epidemiological Investigation” used the funds the team received for the prototype nextstrain.org as a proof of concept to help them secure NIH funding from 2 unrelated NIH funding lines in addition to the OSP. However, other teams were still seeking additional funding.

The OSP has implications for organizational policy, technology development, and the funding of science. With respect to organizational funding policies, receiving broad input from not only experts but also from the public can increase interest in and awareness of open data, as well as potentially provide new ideas of how to leverage open data to address research gaps. At the same time, adhering to funding authority rules and regulations is crucial. The model established with the OSP, in which experts and the public provide comments for the funding organizations to consider but the ultimate funding decisions are made by authorized staff, meets both aims. It is also important to consider who owns the technology developed with open data. In this case, the funding organizations encouraged the creation of open source technology but left it to the solvers to establish their intellectual property and business models for the proposed and proof-of-concept technology. Future research is needed to determine which business models for open data technology development are most effective and sustainable over time.

### Lessons learned/recommendations

We believe that the OSP is a useful model that other funders could adopt to help advance global health (and related issues) through the reuse of open data. In taking such ideas forward, however, the following lessons learned may be instructive and helpful:

1. **Partnerships are always more time-consuming than first imagined.** Though the benefits of collaborative working are significant—shared expertise, better reach, ability to leverage additional funding, etc.—funders should enter into these partnerships mindful that meeting the differing requirements/rules/ambitions of a group of funders is not always straightforward, and an ability to compromise and work through issues is key. It is also critical for funding partners to develop a strong shared commitment and understanding of what they are trying to achieve, as it creates a foundation for working through the challenges that arise and forges good personal/working relationships.
2. **A 2-step funding model is an effective way to encourage innovation whilst minimizing the cost.** The benefits are 2-fold: the short-listed applicants receive funds in order to make the best case for support of their prototype, and the funders, judges, and advisors benefit from seeing the progress made between the 2 stages, which can inform the further selection process. However, it could be argued that having Phase I applicants provide detailed specifications of the technology to be developed may not have encouraged more innovative, off-the-wall, or high-risk ideas that if successful could have more impact. How to adapt the funding and review processes to encourage more innovation, particularly from groups outside traditional health research sectors, requires further discussion and deliberation.
3. **Public participation is a good way to increase the reach of the competition and generate interest and enthusiasm for open data.** The nature of this competition means that engagement with and wider use of the tools is critical to their impact. Holding a public vote was an effective way to raise awareness of open science. It allowed a number of tools to be highlighted, not just the overall winner. It is important to strike the right balance when deciding the contribution that public voting makes to the determination of the eventual winner.
4. **Proposals at differing stages of development were received.** Define clearly whether applications have to focus on a completely new concept (or tool) or whether an existing tool/service—which wishes to add additional new functionality—is also eligible. If both are eligible, it is important to consider how to tension the 2 types of applications against one another in the fairest and most transparent way.
5. **Setting a broad remit allowed a wide range of ideas to be proposed, but the 6 finalists naturally did not fully represent the breadth of ideas proposed.** Consider establishing different categories—best new tool, best cross-cutting application, etc.—rather than having a single winner. Alternatively, consider refining the OSP funding model to create future global initiatives focused in particular areas (e.g., gene-by-environment research, rare diseases, immunization, obesity).
6. **The international funding partnership increased both the global reach of the competition as well as the resources available.** Expanding partnerships to include other international funding organizations with common interests in open data could further help to leverage resources and address intractable global health issues.
7. **Furthermore, it is recommended that funders consider ways to incentivize the sustainability of tools and technologies that leverage open biomedical data to improve biomedical research and public health.** We encourage others to explicitly discuss international funding models and the sharing of resources to advance the use of biomedical open data.

## Conclusions

The OSP was useful to all 3 of the funding agencies in that it provided a range of exemplars that successfully demonstrated the benefits of open data for sparking innovation and generating new societal benefits. Prior developer challenges have emphasized the leveraging of open digital content to create innovative tools (e.g., “National Cancer Institute Using Public Data for Cancer Prevention and Control: From Innovation to Impact challenge” and “National Cancer Institute-DREAM drug sensitivity prediction challenge”). The OSP took this concept 1 step further and encouraged international collaborations to solve global health issues by leveraging open digital content. OSP complements other funding initiatives focused on data-driven discovery (e.g., Gordon and Betty Moore Foundation’s Investigators in Data-Driven Discovery Awards or NIH’s Research Project Grant RO3 program awards) by focusing specifically on publicly available open data.

While market intelligence is typically a benefit of challenge competitions, the international aspects of this competition enriched each of the funding agencies’ understanding of new ideas emerging around the globe and of the mechanisms behind that. The 6 solutions that moved from Phase I to Phase II were exemplars of innovative and useful tools/platforms that leverage open data. Moreover, the other 90 entries provided insight into emerging areas of interest, such as community annotation and knowledge-extraction tools. The other way in which the competition was notable was in its ability to highlight the value of open health data to the broader scientific community. As evidenced by the submissions, press coverage, and Twitter (#openscienceprize) views, the OSP garnered much interest from scientists and citizens around the world. The OSP has provided a pathway that agencies dealing with any type of open data can utilize to incentivize innovative, international team–based science.

## Abbreviations

HHMI: Howard Hughes Medical Institute
NIH: National Institutes of Health
OSP: Open Science Prize
WT: Wellcome Trust

## References

[1] McKiernan EC, Bourne PE, Brown CT, Buck S, Kenall A, Lin J, et al How open science helps researchers succeed. Elife. 2016;5. 10.7554/eLife.16800 PMC4973366 27387362

[2] Bjork BC, Solomon D. Open access versus subscription journals: a comparison of scientific impact. BMC Med. 2012;10:73 doi: 10.1186/1741-7015-10-73 22805105 10.1186/1741-7015-10-73 PMC3398850

[3] Hampton SE, Anderson SS, Bagby SC, Gries C, Han X, Hart EM, et al The Tao of open science for ecology. Ecosphere. 2015;6(7):1–13.

[4] Wells TN, Willis P, Burrows JN, Hooft van Huijsduijnen R. Open data in drug discovery and development: lessons from malaria. Nat Rev Drug Discov. 2016 10;15(10):661–2. doi: 10.1038/nrd.2016.154 27516171 10.1038/nrd.2016.154

[5] National Institutes of Health. NIH-Wide Strategic Plan: Fiscal Years 2016–2020. Turning Discovery Into Health 2016. [cited 2017 Mar 20] Available from: https://www.nih.gov/sites/default/files/about-nih/strategic-plan-fy2016-2020-508.pdf.

[6] Wellcome Trust. Open Research 2016. [cited 2017 Mar 20] Available from: https://wellcome.ac.uk/what-we-do/our-work/open-research.

[7] Howard Hughes Medical Institute. Public access to publications 2007 [cited 2017 Mar 20] Available from: http://www.hhmi.org/sites/default/files/About/Policies/sc320-public-access-to-publications.pdf.

[8] Howard Hughes Medical Institute. Research policies: Sharing published materials/responsibilities of HHMI authors (SC-300) 2015. [cited 2017 Mar 20] Available from: http://www.hhmi.org/sites/default/files/About/Policies/sc_300.pdf.

[9] H.R.5116—America Creating Opportunities to Meaningfully Promote Excellence in Technology, Education, and Science Reauthorization Act of 2010, (2010). [cited 2017 Mar 20] Available from: https://www.gpo.gov/fdsys/pkg/PLAW-111publ358/pdf/PLAW-111publ358.pdf.

[10] National Human Genome Research Institute. Calculating the economic impact of the Human Genome Project 2013. [cited 2017 Mar 20] Available from: https://www.genome.gov/27544383/calculating-the-economic-impact-of-the-human-genome-project/.

[11] Lakhani KR, Boudreau KJ, Loh PR, Backstrom L, Baldwin C, Lonstein E, et al Prize-based contests can provide solutions to computational biology problems. Nat Biotechnol. 2013;31(2):108–11. doi: 10.1038/nbt.2495 23392504 10.1038/nbt.2495 PMC4527172

[12] Chan L, Kirsop B, Arunachalam S. Towards open and equitable access to research and knowledge for development. PLoS Med. 2011;8:3:e1001016 doi: 10.1371/journal.pmed.1001016 21483470 10.1371/journal.pmed.1001016 PMC3066136

